# Crystalline geological bedrock headwater stream contamination in an agricultural-extensive rural watershed: keys factors for the behaviour of neutral and ionic pesticides using a passive sampling approach

**DOI:** 10.1007/s11356-025-36378-4

**Published:** 2025-04-12

**Authors:** Robin Guibal, Julie Leblanc, Karine Cleries, Rachel Martins de Barros, Matthias Monneron-Gyurits, Yoann Brizard, Sophie Lissalde, Gilles Guibaud

**Affiliations:** 1 University of Limoges, E2Lim UR 24 133, 123 Avenue Albert Thomas, 87060 Limoges Cedex, France; 2 Ecométrique, 1 Avenue d’Ester, 87100 Limoges, France; 3 SABV, Syndicat d’Aménagement du Bassin de La Vienne, 38 Avenue du Président Wilson, 87700 Aixe-Sur-Vienne, France

**Keywords:** Passive sampling, POCIS, Organic pollutants, Headwater stream, Neutral and ionic pesticides

## Abstract

The Aixette Watershed, located in southwestern France, was semi-continuously monitored (every 14 days) over 3 years at four different sampling spots with Polar Organic Chemical Integrative Samplers (POCIS), using the Oasis® HLB configuration (for 64 neutral pesticides/metabolites) and the Oasis® MAX configuration (for 15 ionic pesticides/metabolites). This watershed is characterised by extensive agricultural practices (bovine and ovine production) with breeding areas and cereal growing for cattle feeding. The downstream of the Aixette River is located in a peri-urban area. The neutral pesticide contamination could be qualified as background contamination noise, whereas ionic pesticide contamination revealed contamination peaks. The detection frequencies for neutral compounds were higher than those for ionic compounds. Different contamination behaviours were shown by a wider distribution of the time-weighted average concentration (TWAC) obtained for the ionic compounds (median and average TWAC were significantly different). The difference in the trends between neutral and ionic compounds could be explained by their large affinity to water, in accordance with their solubility or logP. Nevertheless, the trend of ionic compounds could not be linked to rainfall but to the flowrate of the Aixette river. This means than contamination with ionic pesticides or metabolites is mainly linked to drainage water from groundwater compared to runoff water, which is consistent with the hydrogeologic behaviour on crystalline geological bedrock and the leaching of ionic compounds from the surface to the aquifer.

**Supplementary Information:**

The online version contains supplementary material available at 10.1007/s11356-025-36378-4.

## Introduction

The presence of organic micropollutants in the environment, such as pesticides and their by-products, has been reported previously (Guibal et al. 2018b; Sousa et al. 2018). Human activities such as industrial production or agriculture may explain this occurrence by the transfer from their application areas to freshwater. There is a wide variety of pesticide families with different phytosanitary actions, which implies that pesticides have different chemical natures. These different natures confer them different physicochemical properties (i.e., solubility, hydrophobicity, affinity for soils, half-life) and, consequently, different behaviours in the environment. For example, some pesticides have short half-lives (< 20 days) and are therefore considered non-persistent in the environment. These pesticides are detectable or quantifiable only through their degradation compounds (i.e. metabolites), which, however, do not have the same chemical properties as the parent compounds.

To monitor contamination levels, monitoring programmes have been implemented in different contexts and scales. For example, the Water Framework Directive (WFD) (2000/60/EC and 2013/39/EC) is applied on a European scale for the monitoring of 18 neutral pesticides in water, such as atrazine and diuron. Nevertheless, no ionic compounds are currently monitored, despite their use in agriculture.

To better understand the different chemical classes, it is important to know their trajectory at the watershed scale. In this context, the use of passive samplers, such as the polar organic chemical integrative sampler (POCIS), is interesting. Passive samplers are deployed over periods of few days to several weeks (usually 14 days for POCIS Alvarez et al. 2004; Mazzella et al. 2010)), and the continuous accumulation of organic compounds provides a time-weighted average concentration (TWAC)(Vrana et al. 2005). For the monitoring of polar organic pesticides, the POCIS with Oasis® HLB as receiving phase is commonly used (Alvarez et al. 2004). More recently, the receiving phase Oasis® MAX was used to sample the ionic compounds in POCIS (Fauvelle et al. 2012), but this configuration was never used at the watershed scale.

The objectives of this study were to monitor the contamination of neutral and ionic pesticides and their metabolites in four sampling sites in rivers during a 3-year semi-continuous study. The watershed is located on a crystalline bedrock (granite and gneiss), where groundwater present in alterites plays a major role in river water supply when the watershed is weakly favorable to run off water. For that, two POCIS configurations were used: the conventional one with Oasis® HLB (POCIS-HLB) for neutral compounds and the configuration with the Oasis® MAX (POCIS-MAX) for ionic compounds. The data acquired throughout these 3 years of monitoring allowed to compare the contamination of two groups of pesticides and metabolites (neutral versus ionic) and to discuss the climatic, geologic or use factors that influence the presence of these compounds in water and, if necessary, the periods during which the use of natural water is linked to contamination risks.

## Materials and methods

### Materials and reagents

HPLC–MS grade solvents were obtained from Carlo Erba (methanol and formic acid), Sigma Aldrich (etyl acetate), and Scharlau (ammonium formate). Ultrapure water (18.2 MΩ.cm) was produced using a Milli-Q Gradient A10 system from Millipore. Neutral and ionic pesticides (64 neutral, 18 ionic, including metabolites—see Table S1) were purchased from Dr Ehrenstorfer (HPLC grade—see Table S2). Internal standards (deuterated pesticides) and their associations are detailed in Tables S3 and S4.

### POCIS (HLB & MAX) preparation and extraction

Two POCIS types were used with 200 mg of Oasis® HLB or Oasis® MAX, between two polyethersulfone membranes (90 mm, 0.1 µm, Pall Supor®) previously washed according to Guibal et al. (Guibal et al. 2015). After deployment, the receiving phase was transferred to pre-weighed SPE cartridges, dried under nitrogen, and stored at − 18 °C during maximum 6 months.

The POCIS-HLB elution was performed by the successive addition of 3 mL of methanol and 3 mL of a 75/25 (v/v) mixture of methanol and ethyl acetate. The POCIS-MAX elution was performed by the addition of 5 mL of a 75/25 (v/v) mixture of methanol and ethyl acetate, followed by 5 mL of a 90/10 (v/v) mixture of methanol and formic acid (1 M). The eluates were evaporated to dryness and then reconstituted in 1 mL of methanol and spiked with internal standards (final concentration: 100 µg L^−1^).

### Calculation of time-weighted average concentration with POCIS

The POCIS is considered an integrative sampler and typically used during the linear sorption phase. Compound TWAC in water can be calculated by the following equation (Alvarez et al. 2004; Mazzella et al. 2007):

$$TWAC=\frac{{C}_{POCIS}\times {m}_{sorbent}}{Rs\times t}$$

where C_POCIS_ the compound concentration in the receiving phase determined after elution and analysis (in µg g^−1^), m_sorbent_ is the therotical sorbent mass (≈ 0.200 g), TWAC is the water compound concentration determined after deployment (in µg mL^−1^), t is the exposure time (in day), and Rs is the sampling rate specific to each compound (in mL d^−1^ – see Tables S3 and S4). In this study, no Performance Reference Compound (PRC) was used.

### Main characteristics of the headwater stream

**Fig. 1:**
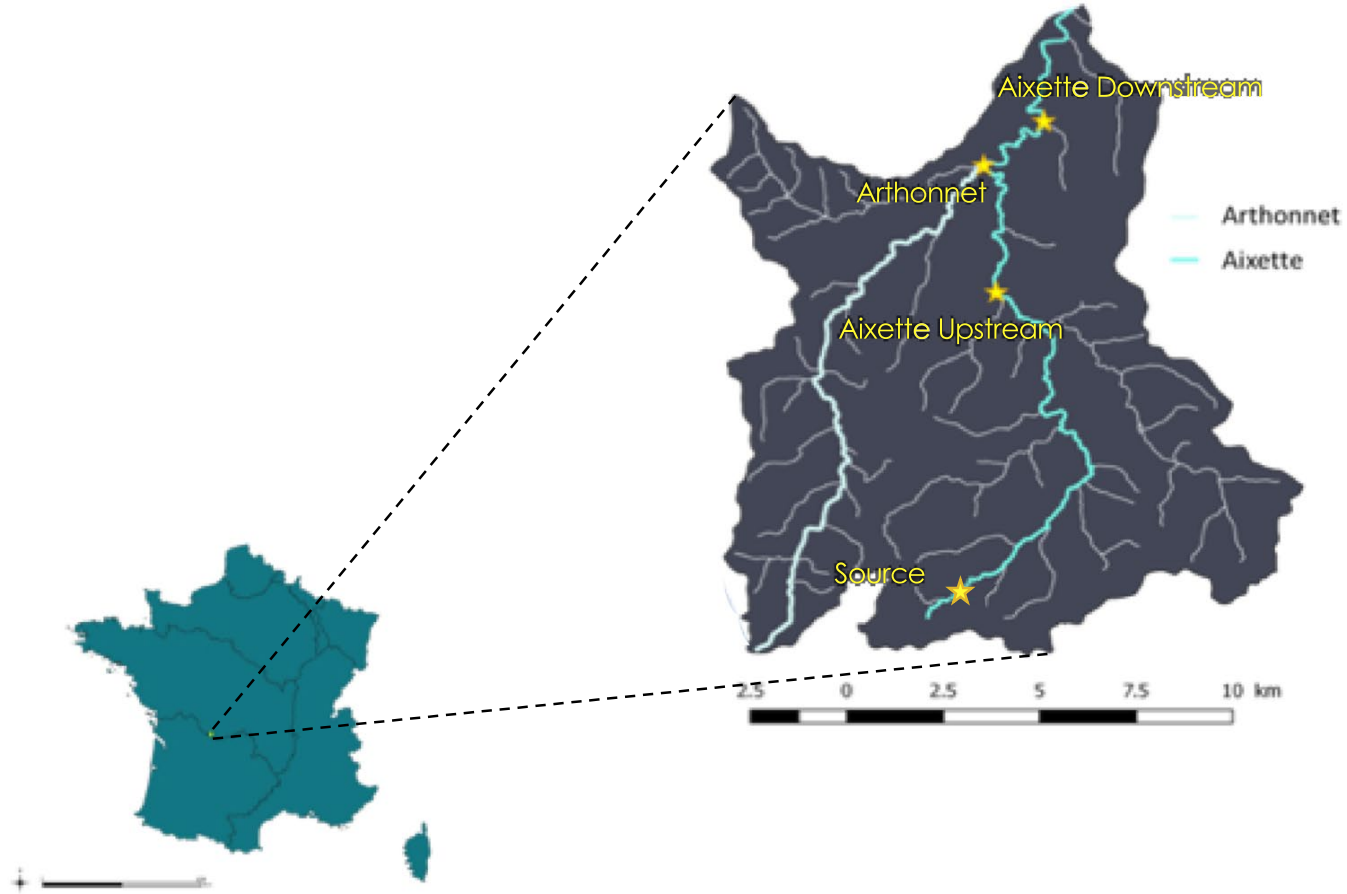
Maps showing the Aixette Watershed and the location of the four sampling points

This study was performed on the watershed of Aixette, a headwater stream in the Haute-Vienne department (southwestern France). This watershed was described in a previous study (Guibal et al. 2018a) and is shown in Fig. 1. This watershed is located on a crystalline bedrock (gneiss and granite) with slight groundwater storage in alterites (depth 1–10 m) and fissured crystalline rocks (depth 10–50 m) (see hydro-system diagram in Fig. S1 and geologic map of the watershed Fig. S2). The climate is oceanic but affected by climate change, mainly in the last 10 years, with a change in rain frequency (annual rainfall is unchanged—Fig. S3 and S4) and a slight increase in the average temperature. The agricultural area (grassland, cattle pastures and cereal fields) represents 63% of the watershed, mainly due to the presence of extensive breeding of Limousin cows and cereal cultivation. The land use is weakly favourable to runoff water. As shown in Fig. 1, this watershed is characterised by a high density of small streams and the presence of wetlands (7.5% of the area) and forests (16%). At the confluence with the Vienne River, the Aixette Watershed is located in an urban area, albeit with a low population density (approximately 50 inhabitants/km^2^).

### POCIS deployment

To monitor pesticide contamination, POCIS devices were deployed at four sites in the Aixette watershed from January 2017 to December 2019 for 14-day periods of deployement ensuring a linear uptake in the POCIS without reaching equilibrium saturation (Alvarez et al. 2004; Li et al. 2010; Morin et al. 2013).

The sampling sites are presented in Fig. 1. The POCIS apparatuses were exposed in plastic nets (POCIS-HLB and POCIS-MAX).

### Instrumental analysis

Analyses were performed using a high-performance liquid chromatograph (1290 Infinity from Agilent Technology) coupled with a high-resolution mass spectrometer (6540 Q-ToF Accurate Mass from Agilent). Positive (for neutral compounds) and negative (for ionic compounds) ionisation modes were operated by an electrospray ionisation source (ESI). Mass acquisition was performed in “all-ions” mode with 0, 10, 20 and 40 eV as collision energies. The source parameters (Tables S5 and S6) and chromatographic separation (Tables S7 and S8) are presented in the Supplementary Materials.

### Quality assurance and control

During analysis, QA/QC were used to control any deviations. The QA/QC consisted of a blank (UPW) and control solutions (50 and 100 µg L^−1^, prepared daily) injection every 10 injections.

To evaluate the contamination of the deployed POCIS during manipulation, storage, transportation and processing, POCIS blanks were performed at each field deployment. These blanks were brought to the sampling sites in aluminum foil like the deployed POCIS. The POCIS blanks were exposed to the air during the manipulation of the deployed POCIS and were stored at 4 °C when the deployed POCIS were exposed in the study environment, i.e. the Aixette River or its tributary. The POCIS blanks and the deployed POCIS were eluted and analysed simultaneously.

## Results and discussion

### Neutral versus ionic pesticide contamination of water

**Fig. 2:**
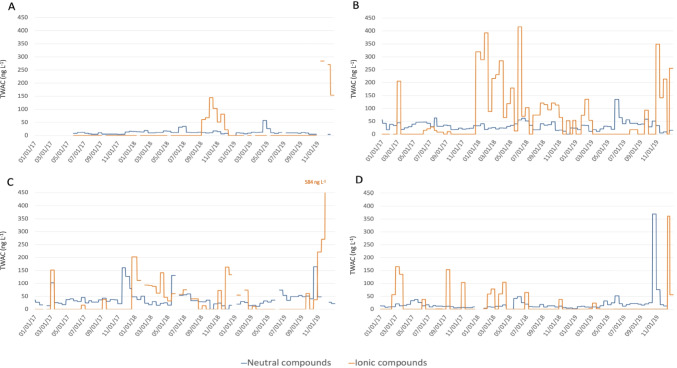
Neutral and ionic pesticide TWAC (ng L^−1^) levels for the Aixette Watershed. **A**: Aixette source (n_POCIS-HLB_ = 61 and n_POCIS-MAX_ = 58), **B**: Aixette upstream (n_POCIS-HLB_ = 77 and n_POCIS-MAX_ = 76), **C**: Aixette downstream (n_POCIS-HLB_ = 72 and n_POCIS-MAX_ = 72) and **D**: Arthonnet tributary (n_POCIS-HLB_ = 73 and n_POCIS-MAX_ = 75)

The TWAC values were determined for each 14-day period of POCIS deployement over the 3 years of monitoring of the four sampling sites. The contamination levels for neutral and ionic compounds (pesticides and metabolites) are shown in Fig. 2 in blue and orange, respectively. These two classes of pesticides were not detected/quantified in the river in the same pattern.

Neutral compounds showed continuous low-level contamination (Total content of compounds monitored < 50 ng L^−1^) throughout the 3 years of monitoring with occasional peaks (Total content of compounds monitored ranging from100 to 350 ng L^−1^) once or twice a year.

Because of the use of POCIS, the neutral compounds could be easily quantified at low concentrations corresponding to “background noise” contamination. These contamination patterns and levels aligns with findings in other headwater streams in France (Guibal et al. 2018b) or small rivers in an agricultural watershed in Midwest U.S. (Van Metre et al. 2017).

In contrast, the average concentrations of ionic compounds showed more variability over time with frequent contamination peaks (more than 10 times per year) and higher than those of the neutral pesticides (in range of 100 to 600 ng L^−1^ in TWAC).

**Fig. 3:**
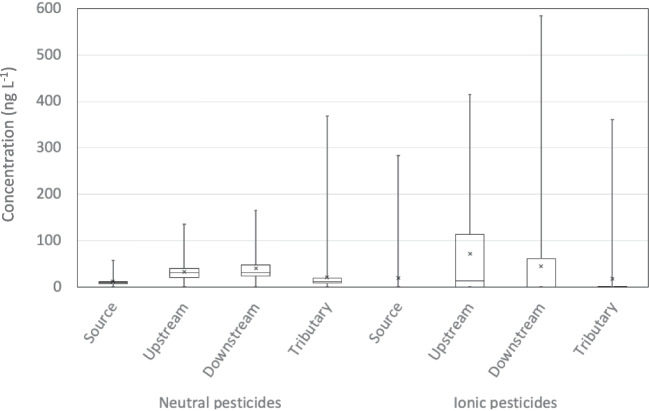
Distribution of TWAC (in ng L^−1^) levels for neutral and ionic pesticides for the four sampling sites (minimum, first quartile, median, third quartile, maximum TWAC: cross is the average TWAC)

Figure 3 shows the TWAC distribution for both pesticide classes detected and quantified during the 3 monitoring years. For neutral pesticides, the median and average TWAC values were similar, indicating similar concentration distributions throughout the 3 years of monitoring. This is consistent with the values reported by (Guibal et al. 2018b) and (Bernard et al. 2019). In contrast, for ionic pesticides, the difference between the median and the average TWAC was higher, indicating that the TWAC varied more significantly throughout the 3 years. These results confirm the different contamination patterns of neutral and ionic pesticides, i.e. a “background noise” contamination of neutral pesticides opposed to strong contamination peaks for ionic pesticides.

For each sampling site, the detection frequencies of the different pesticides (neutral and ionic compounds) and their highest TWAC values are presented and discussed in supplementary materials (Fig. S5). Briefly, the detection frequencies were higher for neutral than for ionic pesticides. Neutral pesticides were often detected and constitute a background noise, whereas ionic pesticides were less frequently detected.

Peaks for neutral and ionic compounds were not detected simultaneously. Neutral pesticides were regularly detected and/or quantified throughout the monitoring period while ionic compounds were detected in the form of contamination peaks. Similar fluctuations have been reported (Farlin et al. 2018) for the metabolites OA and ESA of metolachlor (also investigated in our study), even in an aquifer. These variations may be linked to seasonal pesticide use and the physico-chemical properties of the compounds. In particular, the physico-chemical properties of these two classes of compounds can explain their different behaviours in the environment (see Tables S9 and S10). The neutral compounds detected and/or quantified were polar or moderately polar compounds, with logP values ranging from 0.57 to 3.7 and moderate solubilities (average 3,000 mg L^−1^), whereas the ionic compounds were highly polar, with logP values that could be negative (from − 1.9 to 1.6) and high solubilities (average 66,000 mg L^−1^, with a maximum of 360,000 mg L^−1^ for metolachlor-OA). The half-life values in soil of some neutral pesticides (e.g. s-metolachlor, metazachlor, acetochlor) are less than 25 days, making these compounds non-persistent. The transformation by-products, metabolites, of some of these pesticides do not have the same physico-chemical properties as the parent compounds. This is the case for the metabolites of chloroacetanilides (OA and ESA metabolites of the herbicides metolachlor and acetochlor monitored in our study—see Table S10). The ionic compounds detected and/or quantified in this study were mainly metabolites from neutral pesticides.

**Fig. 4:**
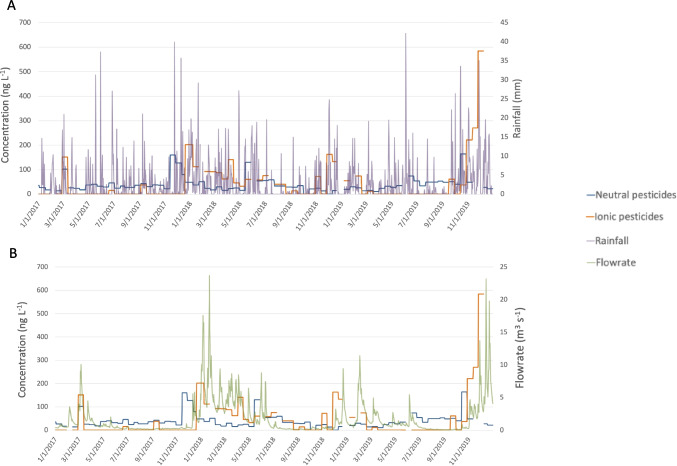
Neutral and ionic compound levels (TWAC in blue and orange, respectively) in Aixette River downstream (in ng L^−1^) and in purple (**A**) and green (**B**), rainfall (in mm) and flowrate (in m^3^ s^−1^) of the watershed (in m^3^ s^−1^)

After treatment, the pesticides end up in the soil, where they can be rapidly transformed into by-products, constituting a stock of pesticides and degradation products. During a rainfall event intense enough to cause leaching, ionic metabolites and compounds with a high affinity for water can reach the aqueous compartment. This results in a delay between the treatment (neutral compounds), the rain event and contamination by ionic pesticides. Figure 4A illustrates the presence of neutral and ionic compounds in the Aixette downstream over 3 years alongside rainfall data. However, no direct correlation between rainfall and neutral or ionic contamination was clearly observed. Because of the complex hydrological context (drought or rainy period, groundwater level), it is not possible to use rainfall as the only indicator to explain the transfer of pesticides or by-products into the aquatic compartment. The flowrate was therefore chosen to highlight the mechanism underlying the transfer of pesticides or metabolites to the aquatic compartment (Fig. 4B) in the context of a geological bedrock with a slight ability of groundwater storage. The behaviours of neutral and ionic compounds with respect to the flowrate differed. The contamination peaks of the neutral compounds appeared independent of flowrate fluctuations, whereas those of the ionic compounds seemed to follow the flowrate fluctuations of the Aixette River.

In contrast to rainfall, this new indicator allows to consider the state of the aquifer (saturated or unsaturated zone) and provides information on the transfer level during rainfall events. However, the relationship between rainfall and river flow can indicate the height of the saturated zone in the alterites and fractured rocks. In fact, when the cumulative rainfall is high and the river flowrate does not increase significantly, the water level is low and the aquifer is being recharged. In general, in this situation, the average pesticide concentrations in the river do not increase significantly. It can be assumed that the main source of pesticides is run-off water, with low pesticide levels in the studied area. Given that pesticides are only used on a small proportion of agricultural land (approximately 10%) in the watershed area, the pesticide input into the environment is low. Generally, the aquifer contributes to the river and, therefore, to the input of pesticides and metabolites.

When high rainfall and a sharp flowrate increase, the saturated zone is high and the aquifer, in addition to run-off water, significantly contributes to feeding the river. This results in a sharp increase in the average pollutant concentrations, mainly those of pesticides and ionic metabolites derived from pesticides, as well as the metabolites stored in the weathered and fractured rocks. This release is most likely facilitated by the compounds’ high affinity for water (low logP and solubility). This hypothesis is supported by the fact that some ionic metabolites can contaminate aquifers (Farlin et al. 2018). The monitoring of such compounds in the alterites, from which water for human consumption is collected, confirms the presence of these compounds in groundwater above crystalline geologic bedrocks (Guibal et al. 2023). The concentrations of the ionic compounds found in groundwater are high in alterites when the saturated zone is high because of considerable rainfall episodes. In dry periods, when the saturated zone rapidly decreases in the alterites, the concentrations of compounds in the groundwater decrease.

## Conclusion

Irrespective of the sampling site, throughout the 3 years of monitoring via passive sampling using two types of POCIS devices (HLB and MAX), the neutral and ionic pesticides presented different trends regarding their levels in the river water. Contamination by neutral pesticides was observed as “background noise” contamination, whereas the contamination by ionic pesticides was the peak contamination. This “background noise” contamination was always detected for all four sampling sites (with two peaks in each year), whereas peak contamination by ionic pesticides was detected periodically. Contamination with ionic compounds was related to the river flowrate which seems consistent considering their physicochemical constants (logP < 2 and high solubility) but also the river supply by aquifer on a crystallin bedrock. The Aixette River was contaminated by these ionic compounds at times of significant rainfall accumulation, allowing the compounds stored in the alterites and fractured bedrock to be transferred to the river via the aquifers.

## Supplementary Information

Below is the link to the electronic supplementary material.

Supplementary file1 (DOCX 3168 KB)

## Data Availability

All data are available in the manuscript or in the supplemantary materials.
