# Supplementary material for: Crystalline geological bedrock headwater stream contamination in an agricultural-extensive rural watershed: keys factors for the behaviour of neutral and ionic pesticides using a passive sampling approach
Source: Environ Sci Pollut Res Int. 2025 Apr 12;32(37):21870–6. doi: 10.1007/s11356-025-36378-4 (PMC12488814; doi:10.1007/s11356-025-36378-4)
Supplement: Supplementary file 1 — Supplementary file1 (DOCX 3168 KB) [file 11356_2025_36378_MOESM1_ESM.docx]

**Crystalline geological bedrock headwater stream contamination in an agricultural-extensive rural watershed: keys factors for the behaviour of neutral and ionic pesticides using a passive sampling approach**

**SUPPLEMENTARY MATERIALS**

Robin Guibal^1*^, Julie Leblanc^1^, Karine Cleries^1^, Rachel Martins de Barros^1^, Matthias Monneron-Gyurits^1,2^, Yoann Brizard^3^, Sophie Lissalde^3^, Gilles Guibaud^4^

(1) University of Limoges, E2Lim UR 24 133, 123 Avenue Albert Thomas, 87060 Limoges Cedex, France

(2) Ecométrique, 1 Avenue d’Ester, 87100 Limoges, France

(3) SABV, Syndicat d’Aménagement du Bassin de la Vienne, 38 Avenue du Président Wilson, 87700 Aixe-sur-Vienne, France

* Corresponding author: robin.guibal@unilim.fr

Table S1: Main data of the neutral pesticides and compounds studied in this study

| **Neutral pesticides** | ***CAS Number*** | ***Chemical formula*** | ***Molar weight (g mol^-1^)*** | ***Purity (%)*** | ***Manufacturer*** |
| --- | --- | --- | --- | --- | --- |
| 3-hydroxy-carbofuran | 16655-82-6 | C_12_H_15_NO_4_ | 237.25 | 99.0 | Dr. Ehrenstorfer |
|  |  |  |  | 99.8 | HPC Standarts GmbH |
| Acetochlor | 34256-82-1 | C_14_H_20_ClNO_2_ | 269.8 | 98.0 | Dr. Ehrenstorfer |
| Alachlor | 15972-60-8 | C_14_H_20_ClNO_2_ | 269.77 | 99.5 | Dr. Ehrenstorfer |
| Atrazine | 1912-24-9 | C_8_H_14_ClN_5_ | 215.69 | 99.0 | Dr. Ehrenstorfer |
| Azoxystrobin | 131860-33-8 | C_22_H_17_N_3_O_5_ | 403.39 | 99.5 | Dr. Ehrenstorfer |
| Benoxacor | 98730-04-2 | C_11_H_11_Cl_2_NO_2_ | 260.1 | 99.5 | Dr. Ehrenstorfer |
| Carbaryl | 63-25-2 | C_12_H_11_NO_2_ | 201.23 | 98.9 | Dr. Ehrenstorfer |
| Carbendazim | 10605-21-7 | C_9_H_9_N_3_O_2_ | 191.19 | 99.5 | Dr. Ehrenstorfer |
| Carbofuran | 1563-66-2 | C_12_H_15_NO_3_ | 221.26 | 99.0 | Dr. Ehrenstorfer |
| Chlorfenvinphos | 470-90-6 | C_12_H_14_Cl_3_O_4_P | 359.57 | 99.0 | Dr. Ehrenstorfer |
| Chlorpyriphos | 2921-88-2 | C_9_H_11_Cl_3_NO_3_PS | 350.59 | 98.5 | Dr. Ehrenstorfer |
| Chlortoluron | 15545-48-9 | C_10_H_13_ClN_2_O | 212.7 | 99.0 | Dr. Ehrenstorfer |
| Cyanazine | 21725-46-2 | C_9_H_13_ClN_6_ | 240.69 | 98.0 | Dr. Ehrenstorfer |
| Cybutrine (Irgarol) | 28159-98-0 | C_11_H_19_N_5_S | 310.47 | 99.0 | Dr. Ehrenstorfer |
| Cyproconazole | 94361-06-5 | C_15_H_18_ClN_3_O | 291.78 | 99.0 | Dr. Ehrenstorfer |
| Cyromazine | 66215-27-8 | C_6_H_10_N_6_ | 166.18 | 98.0 | Dr. Ehrenstorfer |
| DCPMU | 3567-62-2 | C_8_H_8_Cl_2_N_2_O | 219.07 | 97.7 | Dr. Ehrenstorfer |
| DCPU | 2327-02-8 | C_7_H_6_Cl_2_N_2_O | 205.04 | 98.5 | Dr. Ehrenstorfer |
| DEA | 6190-65-4 | C_6_H_10_ClN_5_ | 187.63 | 99.0 | Dr. Ehrenstorfer |
| DIA | 1007-28-9 | C_5_H_8_ClN_5_ | 173.61 | 98.7 | Dr. Ehrenstorfer |
| Dichlorvos | 62-73-7 | C_4_H_7_Cl_2_O_4_P | 221.98 | 99.9 | HPC Standarts GmbH |
| Diflufenicanil | 83164-33-4 | C_19_H_11_F_5_N_2_O_2_ | 394.3 | 98.8 | Dr. Ehrenstorfer |
| Dimethachlor | 50563-36-5 | C_13_H_18_ClNO_2_ | 255.74 | 98.0 | Dr. Ehrenstorfer |
| Dimethenamid | 87674-68-8 | C_12_H_18_ClNO_2_S | 275.79 | 99.0 | Dr. Ehrenstorfer |
| Dimethoate | 60-51-5 | C_5_H_12_NO_3_PS_2_ | 229.28 | 98.0 | Dr. Ehrenstorfer |
| Dimethomorph | 1104188-70-5 | C_21_H_22_ClNO_4_ | 387.86 | 99.0 | Dr. Ehrenstorfer |
| Diuron | 330-54-1 | C_9_H_10_Cl_2_N_2_O | 233.1 | 98.0 | Dr. Ehrenstorfer |
| Epoxiconazole | 106325-08-0 | C_17_H_13_ClFN_3_O | 329.76 | 98.5 | Dr. Ehrenstorfer |
| Ethidimuron | 30043-49-3 | C_7_H_12_N_4_O_3_S_2_ | 264.33 | 99.0 | Dr. Ehrenstorfer |
| Flazasulfuron | 104040-78-0 | C_13_H_12_F_3_N_5_O_5_S | 407.33 | 99.4 | Dr. Ehrenstorfer |
| Flurochloridone | 61213-25-0 | C_12_H_10_Cl_2_F_3_NO | 312.12 | 99.0 | Dr. Ehrenstorfer |
| Flurtamone | 96525-23-4 | C_18_H_14_F_3_NO_2_ | 333.3 | 98.3 | Dr. Ehrenstorfer |
|  |  |  |  | 99.9 | HPC Standarts GmbH |
| Flusilazol | 85509-19-9 | C_16_H_15_F_2_N_3_Si | 315.4 | 96.5 | Dr. Ehrenstorfer |
| Hexazinone | 51235-04-2 | C_12_H_20_N_4_O_2_ | 252.32 | 96.0 | Dr. Ehrenstorfer |
| Imidacloprid | 138261-41-3 | C_9_H_10_ClN_5_O_2_ | 255.66 | 98.0 | Dr. Ehrenstorfer |
| IPPMU | 34123-57-4 | C_11_H_16_N_2_O | 192.26 | 99.5 | Dr. Ehrenstorfer |
| IPPU | 56046-17-4 | C_10_H_14_N_2_O | 178.23 | 99.0 | Dr. Ehrenstorfer |
| Isoproturon | 34123-59-6 | C_12_H_18_N_2_O | 206.29 | 99.0 | Dr. Ehrenstorfer |
| Kresoxim-methyl | 143390-89-0 | C_18_H_19_NO_4_ | 313.36 | 98.5 | Dr. Ehrenstorfer |
| Linuron | 990-55-2 | C_9_H_10_Cl_2_N_2_O_2_ | 249.1 | 99.5 | Dr. Ehrenstorfer |
| Metazachlor | 67129-08-2 | C_14_H_16_ClN_3_O | 277.76 | 98.5 | Dr. Ehrenstorfer |
| Methomyl | 16752-77-5 | C_5_H_10_N_2_O_2_S | 162.21 | 99.5 | Dr. Ehrenstorfer |
| S-Metolachlor | 87392-12-9 | C_15_H_22_ClNO_2_ | 283.79 | 98.0 | Dr. Ehrenstorfer |
|  |  |  |  | 99.7 | HPC Standarts GmbH |
| Metoxuron | 19937-59-8 | C_10_H_13_ClN_2_O_2_ | 228.68 | 99.5 | Dr. Ehrenstorfer |
| Monolinuron | 1746-81-2 | C_9_H_11_ClN_2_O_2_ | 214.65 | 98.2 | HPC Standarts GmbH |
| Norflurazon | 27314-13-2 | C_12_H_9_ClF_3_N_3_O | 303.7 | 94.0 | Dr. Ehrenstorfer |
| Norflurazon-desmethyl | 23576-24-1 | C_11_H_7_ClF_3_N_3_O | 289.67 | 99.0 | Dr. Ehrenstorfer |
| Oxadiazon | 19666-30-9 | C_15_H_18_Cl_2_N_2_O_3_ | 345.23 | 99.0 | Dr. Ehrenstorfer |
| Pendimethalin | 40487-42-1 | C_13_H_19_N_3_O_4_ | 281.31 | 99.0 | Dr. Ehrenstorfer |
| Pirimicarb | 23103-98-2 | C_11_H_18_N_4_O_2_ | 238.29 | 98.7 | Dr. Ehrenstorfer |
| Procymidone | 32809-16-8 | C_13_H_11_Cl_2_NO_2_ | 284.1 | 98.0 | Dr. Ehrenstorfer |
| Propiconazole | 60207-90-1 | C_15_H_17_Cl_2_N_3_O_2_ | 342.22 | 99.9 | HPC Standarts GmbH |
| Prosulfocarb | 52888-80-9 | C_14_H_21_NOS | 251.39 | 98.0 | Dr. Ehrenstorfer |
| Prosulfuron | 94125-34-5 | C_15_H_16_F_3_N_5_O_4_S | 419.38 | 98.5 | Dr. Ehrenstorfer |
| Pymetrozine | 123312-89-0 | C_10_H_11_N_5_O | 217.2 | 99.5 | Dr. Ehrenstorfer |
| Quinoxyfen | 124495-18-7 | C_15_H_8_Cl_2_FNO | 308.14 | 99.9 | HPC Standarts GmbH |
| Simazine | 122-34-9 | C_7_H_12_ClN_5_ | 201.66 | 98.0 | Dr. Ehrenstorfer |
| Spiroxamine | 118134-30-8 | C_18_H_35_NO_2_ | 297.48 | 99.0 | Dr. Ehrenstorfer |
| Tebuconazole | 107534-96-3 | C_16_H_22_ClN_3_O | 307.8 | 98.5 | Dr. Ehrenstorfer |
|  |  |  | 307.82 | 99.9 | HPC Standarts GmbH |
| Terbuthylazine | 5915-41-3 | C_9_H_16_ClN_5_ | 229.72 | 98.5 | Dr. Ehrenstorfer |
| Terbuthylazine-2-hydroxy | 66753-07-9 | C_9_H_17_NO_5_ | 211.26 | 99.4 | HPC Standarts GmbH |
| Terbuthylazine-desethyl | 30125-63-4 | C_7_H_12_ClN_5_ | 201.66 | 99.5 | Dr. Ehrenstorfer |
| Terbutryn | 886-50-0 | C_10_H_19_N_5_S | 241.36 | 99.8 | HPC Standarts GmbH |
| Thiodicarb | 59669-26-0 | C_10_H_18_N_4_O_4_S_3_ | 354.46 | 99.0 | Dr. Ehrenstorfer |

Table S2: Main data of the ionic compounds studied in this study

| **Ionic pesticides** | ***CAS Number*** | ***Chemical formula*** | ***Molar weight (g mol^-1^)*** | ***Purity (%)*** | ***Fabricant*** |
| --- | --- | --- | --- | --- | --- |
| 2.4-D | 94-75-7 | C_8_H_6_Cl_2_O_3_ | 221.04 | 98.0 | Dr. Ehrenstorfer |
| Acetochlor-ESA | 947601-84-5 | C_14_H_20_NNaO_5_S | 337.37 | 96.0 | Dr. Ehrenstorfer |
|  |  |  |  | 98.9 | HPC Standarts GmbH |
| Acetochlor-OA | 194992-44-4 | C_14_H_19_NO_4_ | 265.3 | 98.5 | Dr. Ehrenstorfer |
|  |  |  |  | 99.3 | HPC Standarts GmbH |
| Bentazone | 25057-89-0 | C_10_H_12_N_2_O_3_S | 240.28 | 99.0 | Dr. Ehrenstorfer |
| Chlorsulfuron | 64902-72-3 | C_12_H_12_ClN_5_O_4_S | 357.77 | 97.0 | Dr. Ehrenstorfer |
| Dichlorprop | 120-36-5 | C_9_H_8_Cl_2_O_3_ | 235.07 | 99.5 | Dr. Ehrenstorfer |
| Diclofop | 40843-25-2 | C_15_H_12_Cl_2_O_4_ | 327.17 | 98.0 | Dr. Ehrenstorfer |
| Fenoprop | 97-72-1 | C_9_H_7_Cl_3_O_3_ | 269.51 | 97.0 | Dr. Ehrenstorfer |
| Iodosulfuron | 144550-36-7 | C_14_H_13_IN_5_NaO_6_S | 529.2 | 99.0 | Dr. Ehrenstorfer |
| Ioxynil | 1689-83-4 | C_7_H_3_I_2_NO | 370.92 | 99.0 | Dr. Ehrenstorfer |
| MCPA | 94-74-6 | C_9_H_9_ClO_3_ | 200.62 | 98.5 | Dr. Ehrenstorfer |
| Mecoprop | 93-65-2 | C_10_H_11_ClO_3_ | 214.65 | 98.7 | Dr. Ehrenstorfer |
| Mesotrione | 104206-82-8 | C_14_H_13_NO_7_S | 339.32 | 99.0 | Dr. Ehrenstorfer |
| Metolachlor-ESA | 947601-85-6 | C_15_H_22_NNaO_5_S | 351.41 | 98.0 | Dr. Ehrenstorfer |
|  |  |  | 351.39 | 99.9 | HPC Standarts GmbH |
| Metolachlor-OA | 152019-73-3 | C_15_H_21_NO_4_ | 279.33 | 98.0 | Dr. Ehrenstorfer |
|  |  |  |  | 99.1 | Dr. Ehrenstorfer |
| Metsulfuron-methyl | 74223-64-6 | C_14_H_15_N_5_O_6_S | 381.36 | 99.5 | Dr. Ehrenstorfer |
| Nicosulfuron | 111991-09-4 | C_15_H_18_N_6_O_6_S | 410.4 | 98.0 | Dr. Ehrenstorfer |
|  |  |  | 410.41 | 99.9 | HPC Standarts GmbH |
| Sulcotrione | 99105-77-8 | C_14_H_13_ClO_5_S | 328.77 | 98.5 | Dr. Ehrenstorfer |

Table S3: Neutral compounds characteristics after UHPLC separation and mass detection and sampling rates

| **Neutral pesticides** | ***Rs***  ***(mL day^-1^)*** | ***Retention time (min)*** | ***Accurate Mass (g mol***^-1^) | ***Internal standard*** |
| --- | --- | --- | --- | --- |
| 3-hydroxy-carbofuran | 197 | 4.68 | 238.1071 | Methomyl-d3 |
| Acetochlor | 333 | 13.59 | 270.1245 | Metolachlor-d6 |
| Alachlor | 345 | 13.65 | 270.1245 | Metolachlor-d6 |
| Atrazine | 283 | 8.54 | 216.1003 | Atrazine-d5 |
| Azoxystrobin | 336 | 11.30 | 404.1228 | Atrazine-d5 |
| Benoxacor | 367 | 9.96 | 260.0240 | Diuron-d6 |
| Carbaryl | 169 | 7.33 | 202.0863 | Carbaryl-d3 |
| Carbendazim | 304 | 3.89 | 192.0768 | Methomyl-d3 |
| Carbofuran | 425 | 6.73 | 222.1125 | Carbofuran-d3 |
| Chlorfenvinphos | 278 | 16.03 and 16.50 | 358.9752 | Chlorpyrifos-d5 |
| Chlorpyriphos | 125 | 18.12 | 349.9336 | Chlorpyrifos-d5 |
| Chlortoluron | 251 | 8.30 | 213.0789 | Diuron-d6 |
| Cyanazine | 320 | 6.00 | 241.0963 | Atrazine-d5 |
| Cybutryne (Irgarol) | 238 | 12.15 | 254.1435 | Atrazine-d5 |
| Cyproconazole | 316 | 12.40 | 292.1199 | Tebuconazole-d6 |
| Cyromazine | 200 | 1.67 | 167.1040 | Methomyl-d3 |
| DCPMU | 356 | 8.95 | 219.0086 | Diuron-d6 |
| DCPU | 431 | 7.95 | 204.9930 | Diuron-d6 |
| DEA | 305 | 5.12 | 188.0697 | DEA-d6 |
| DIA | 276 | 4.22 | 174.0541 | DEA-d6 |
| Dichlorvos | 200 | 6.32 | 220.9532 | Carbaryl-d3 |
| Diflufenicanil | 200 | 17.04 | 395.0813 | Chlorpyrifos-d5 |
| Dimethachlor | 292 | 9.53 | 256.1091 | Diuron-d6 |
| Dimethenamid | 287 | 11.17 | 276.0805 | Metolachlor-d6 |
| Dimethoate | 163 | 4.76 | 230.0062 | Methomyl-d3 |
| Dimetomorph | 395 | 11.57 and 12.41 | 388.1301 | Metolachlor-d6 |
| Diuron | 234 | 9.30 | 233.0239 | Diuron-d6 |
| Epoxiconazole | 404 | 14.18 | 330.0806 | Tebuconazole-d6 |
| Ethidimuron | 366 | 4.45 | 265.0424 | DEA-d6 |
| Flazasulfuron | 222 | 10.43 | 408.0584 | Diuron-d6 |
| Flurochloridone | 200 | 12.82 | 312.0165 | Metolachlor-d6 |
| Flurtamone | 360 | 11.30 | 334.1038 | Diuron-d6 |
| Flusilazole | 437 | 14.84 | 316.1061 | Tebuconazole-d6 |
| Hexazinone | 288 | 6.67 | 253.1659 | Atrazine-d5 |
| Imidacloprid | 290 | 4.42 | 256.0596 | Carbofuran-d3 |
| IPPMU | 349 | 8.54 | 193.1335 | Diuron-d6 |
| IPPU | 362 | 7.65 | 179.1179 | Diuron-d6 |
| Isoproturon | 316 | 8.95 | 207.1492 | Diuron-d6 |
| Kresoxim-methyl | 200 | 15.12 | 314.1387 | Tebuconazole-d6 |
| Linuron | 306 | 10.86 | 249.0184 | Diuron-d6 |
| Metazachlor | 289 | 8.74 | 278.1046 | Metolachlor-d6 |
| Methomyl | 306 | 3.70 | 163.0536 | Methomyl-d3 |
| Metolachlor | 338 | 13.95 | 284.1407 | Metolachlor-d6 |
| Metoxuron | 274 | 5.73 | 229.0738 | Diuron-d6 |
| Monolinuron | 280 | 7.62 | 215.0582 | Diuron-d6 |
| Norflurazon | 285 | 9.53 | 304.0458 | Diuron-d6 |
| Norflurazon-desmethyl | 284 | 8.30 | 290.0288 | Diuron-d6 |
| Oxadiazon | 200 | 17.78 | 345.0767 | Chlorpyrifos-d5 |
| Pendimethalin | 260 | 18.10 | 282.1448 | Chlorpyrifos-d5 |
| Pirimicarb | 285 | 5.97 | 239.1503 | Pirimicarb-d6 |
| Procymidone | 380 | 9.43 | 284.0240 | Diuron-d6 |
| Propiconazole | 172 | 15.87 | 342.0771 | Tebuconazole-d6 |
| Prosulfocarb | 200 | 17.29 | 252.1417 | Chlorpyrifos-d5 |
| Prosulfuron | 240 | 11.40 | 420.0948 | Diuron-d6 |
| Pymetrozine | 320 | 3.18 | 218.1036 | Atrazine-d5 |
| Quinoxyfen | 200 | 17.95 | 308.0040 | Chlorpyrifos-d5 |
| Simazine | 281 | 6.70 | 202.0854 | Atrazine-d5 |
| Spiroxamine | 475 | 10.65 | 298.2741 | Diuron-d6 |
| Tebuconazole | 351 | 15.73 | 308.1524 | Tebuconazole-d6 |
| Terbuthylazine | 488 | 11.30 | 230.1155 | Atrazine-d5 |
| Terbuthylazine-2-hydroxy | 292 | 4.59 | 212.1506 | Atrazine-d5 |
| Terbuthylazine-desethyl | 290 | 7.26 | 202.0855 | Atrazine-d5 |
| Terbutryn | 324 | 11.27 | 242.1434 | Diuron-d6 |
| Thiodicarb | 168 | 8.12 | 355.0551 | Pirimicarb-d6 |

Table S4: Ionic compounds characteristics after UHPLC separation and mass detection and sampling rates

| **Ionic pesticides** | ***Rs***  ***(mL day^-1^)*** | ***Retention time (min)*** | ***Accurate Mass (g mol***^-1^) | ***Internal standard*** |
| --- | --- | --- | --- | --- |
| 2.4-D | 130 | 3.73 | 218.9621 | MCPA-d3 |
| Acetochlor-ESA | 76 | 4.05 | 314.1068 | MCPA-d3 |
| Acetochlor-OA | 76 | 3.75 | 264.1241 | MCPA-d3 |
| Bentazone | 171 | 2.34 | 239.0489 | Bentazone-d6 |
| Chlorsulfuron | 94 | 2.67 | 356.023 | Metsulfuron-methyl-d3 |
| Dichlorprop | 136 | 4.76 | 232.9778 | MCPA-d3 |
| Diclofop | 166 | 7.22 | 325.0040 | MCPA-d3 |
| Fenoprop | 152 | 5.20 | 266.9388 | MCPA-d3 |
| Iodosulfuron | 100 | 3.23 | 505.9637 | Metsulfuron-methyl-d3 |
| Ioxynil | 266 | 3.17 | 369.8231 | MCPA-d3 |
| MCPA | 148 | 3.88 | 199.0167 | MCPA-d3 |
| Mecoprop | 120 | 3.64 | 213.0325 | MCPA-d3 |
| Mesotrione | 48 | 2.32 | 338.0340 | MCPA-d3 |
| Metolachlor-ESA | 73 | 4.20 | 328.1224 | MCPA-d3 |
| Metolachlor-OA | 73 | 4.36 | 278.1398 | MCPA-d3 |
| Metsulfuron-methyl | 89 | 3.40 | 380.0670 | Metsulfuron-methyl-d3 |
| Nicosulfuron | 63 | 2.57 | 409.0936 | Bentazone-d6 |
| Sulcotrione | 81 | 2.65 | 327.0099 | MCPA-d3 |

Figure S1: Simplified diagram of the hydrographic system (crystallin geological bedrock) studied watershed


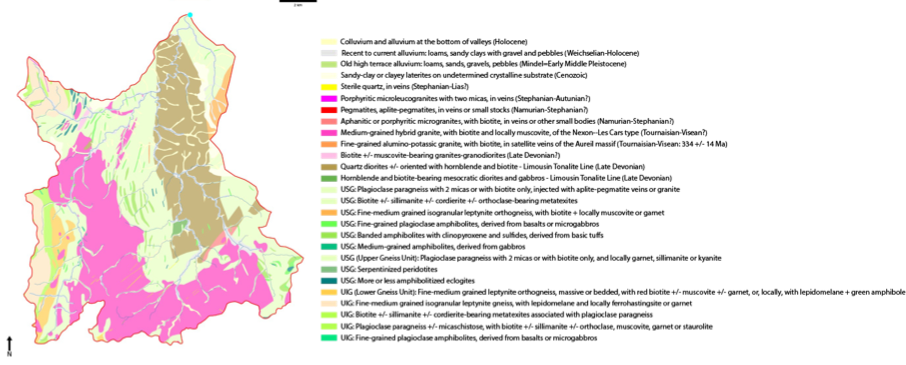


Figure S2: Geologic map of the Aixette watershed

Figure S3: Annual rainfall in Limoges Bellegarde station (infloclimat.fr).

Figure S4: Rainfall and flowrate of Aixette river during the 3 years monitoring.

Table S5: UHPLC-TOF-MS operational parameters in positive ESI ion mode for neutral compounds analysis (*Optimized source parameters)

| **Parameter** | **Value** |
| --- | --- |
| Sheath gas temperature* | 375°C |
| Drying gas temperature* | 130°C |
| Drying gas flow* | 13 L min^-1^ |
| Fragmentor voltage* | 120 V |
| Nebulizer pressure* | 35 psi |
| Capillary voltage | 3500 V |
| Skimmer voltage* | 65 V |
| Mass range (m/z) | 100 – 1500 |
| Reference mass (m/z) | 922.0098 |
| Octopole 1 RF* | 750 V |
| Nozzle voltage* | 300 V |

Table S6: UHPLC-TOF-MS operational parameters in negative ESI ion mode for ionic compounds analysis (*Optimized source parameters)

| **Parameter** | **Value** |
| --- | --- |
| Sheath gas temperature* | 400°C |
| Drying gas temperature* | 130°C |
| Drying gas flow* | 13 L min^-1^ |
| Fragmentor voltage* | 130 V |
| Nebulizer pressure* | 20 psi |
| Capillary voltage | 3500 V |
| Skimmer voltage* | 65 V |
| Mass range (m/z) | 100 – 1500 |
| Reference mass (m/z) | 112.9855 and 1033.9881 |
| Octopole 1 RF* | 750 V |
| Nozzle voltage* | 300 V |

**Instrumental analysis**

Chromatographic separation was performed with a Zorbax Eclipse Plus C18 Rapid Resolution High-Definition (RRHD) column (150 mm × 2.1 mm, 1.8 μm) from Agilent for the separation of neutral compounds and a Nucleoshell RP18+ column (100 mm × 3 mm, 2.1 µm) from Macherey–Nagel for ionic compound separation. For both separation methods, the column and autosampler temperatures were maintained at 30°C and 4°C, respectively, and the injection volume was 5 μL. The elution gradients are presented in Tables S7 and S8.

Table S7: Optimized parameters of the UHPLC method for neutral compounds

| **Time (min)** | **UPW (%)** | **MeOH + 5mM ammonium formate and 0.1% formic acid (%)** | **Flow**  **(µL min^-1^)** |
| --- | --- | --- | --- |
| 0 | 90 | 10 | 400 |
| 0.5 | 90 | 10 | 400 |
| 3.5 | 50 | 50 | 400 |
| 14 | 30 | 70 | 400 |
| 17 | 10 | 90 | 400 |
| 20 | 10 | 90 | 400 |

Table S8: Optimized parameters of the UHPLC method for ionic compounds

| **Time (min)** | **UPW with 5mM ammonium formate (%)** | **MeOH with 5mM ammonium formate (%)** | **Flow**  **(µL min^-1^)** |
| --- | --- | --- | --- |
| 0 | 90 | 10 | 400 |
| 0.5 | 90 | 10 | 400 |
| 2 | 50 | 50 | 400 |
| 5 | 45 | 55 | 400 |
| 7 | 10 | 90 | 400 |
| 10 | 10 | 90 | 400 |

Table S9: Physico-chemical properties of neutral compounds

| **Neutral pesticides** | **Log Kow** | **Solubility in water (in mg L^-1^ at 20°C)** | **DT50 (lab in days)** | **DT50 (field in days)** |
| --- | --- | --- | --- | --- |
| 3-hydroxy-carbofuran | 1.45 | 6207 | 0.35 | *n.d.* |
| Acetochlor | 4.14 | 282 | 10.6 | 12.1 |
| Alachlor | 3.09 | 240 | 35 | 14 |
| Atrazine | 2.70 | 35 | 66 | 29 |
| Azoxystrobin | 2.50 | 6.7 | 84.5 | 180.7 |
| Benoxacor | 2.69 | 20 | *n.d.* | *n.d.* |
| Carbaryl | 2.36 | 9.1 | 16 | *n.d.* |
| Carbendazim | 1.48 | 8.0 | 34.3 | 22 |
| Carbofuran | 1.80 | 322 | 12.8 | 14 |
| Chlorfenvinphos | 3.80 | 145 | 37 | 30 |
| Chlorpyriphos | 4.70 | 1.1 | 386 | 27.6 |
| Chlortoluron | 2.50 | 76 | 33.5 | 12.5 |
| Cyanazine | 2.10 | 171 | 16 | *n.d.* |
| Cybutryne (Irgarol) | 3.95 | 7.0 | *n.d.* | *n.d.* |
| Cyproconazole | 3.09 | 93 | 142 | 129 |
| Cyromazine | 0.07 | 13000 | 31.8 | 9.7 |
| DCPMU | 2.09 | 490 | 127 | *n.d.* |
| DCPU | 2.35 | 940 | 7.7 | *n.d.* |
| DEA | 1.51 | 2700 | 170 | 45 |
| DIA | 1.15 | 980 | *n.d.* | *n.d.* |
| Dichlorvos | 1.90 | 18000 | 2 | *n.d.* |
| Diflufenicanil | 4.20 | 0.1 | 94.5 | 64.6 |
| Dimethachlor | 2.17 | 2300 | 6.5 | 3.2 |
| Dimethenamid | 1.89 | 1499 | 12.1 | 15.8 |
| Dimethoate | 0.75 | 25900 | 2.5 | 7.2 |
| Dimetomorph | 2.68 | 29 | 72.7 | 44 |
| Diuron | 2.87 | 36 | 146.6 | 229 |
| Epoxiconazole | 3.30 | 7.1 | 353.5 | 97.7 |
| Ethidimuron | 0.43 | 3000 | *n.d.* | *n.d.* |
| Flazasulfuron | -0.06 | 2100 | 41.2 | 10 |
| Flurochloridone | 3.36 | 22 | 81.4 | 40.6 |
| Flurtamone | 3.24 | 11 | 10.7 | 35.8 |
| Flusilazole | 3.87 | 42 | 427 | 94 |
| Hexazinone | 1.17 | 33000 | 90 | *n.d.* |
| Imidacloprid | 0.57 | 610 | 187 | 174 |
| IPPMU | 2.63 | *n.d.* | 33 | *n.d.* |
| IPPU | 2.16 | *n.d.* | *n.d.* | *n.d.* |
| Isoproturon | 2.50 | 70 | 12 | 23 |
| Kresoxim-methyl | 3.40 | 2.0 | 0.87 | 1 |
| Linuron | 3.00 | 64 | 57.6 | 48 |
| Metazachlor | 2.49 | 450 | 10.8 | 6.8 |
| Methomyl | 0.09 | 55000 | 6.97 | *n.d.* |
| S-metolachlor | 3.05 | 480 | 51.8 | 23.17 |
| Metoxuron | 1.60 | 678 | *n.d.* | 18.5 |
| Monolinuron | 2.20 | 735 | 50 | *n.d.* |
| Norflurazon | 2.45 | 34 | *n.d.* | 225 |
| Norflurazon-desmethyl | 1.72 | *n.d.* | *n.d.* | *n.d.* |
| Oxadiazon | 5.33 | 0.6 | 502 | 165 |
| Pendimethalin | 5.40 | 0.3 | 182.3 | 100.6 |
| Pirimicarb | 1.70 | 3100 | 502 | 165 |
| Procymidone | 3.30 | 2.5 | 784.5 | 208.3 |
| Propiconazole | 3.72 | 150 | 71.8 | 35.2 |
| Prosulfocarb | 4.48 | 13 | 11.9 | 9.8 |
| Prosulfuron | 1.50 | 4000 | 62.1 | 11.9 |
| Pymetrozine | -0.19 | 270 | 4.6 | 22.6 |
| Quinoxyfen | 5.10 | 0.1 | 308 | 169.3 |
| Simazine | 2.30 | 5.0 | *n.d.* | 90 |
| Spiroxamine | 2.89 | 405 | 22.1 | 52.4 |
| Tebuconazole | 3.70 | 36 | 365 | 47.1 |
| Terbuthylazine | 3.40 | 6.6 | 72 | 21.8 |
| Terbuthylazine-2-hydroxy | *n.d.* | 7.2 | 559 | *n.d.* |
| Terbuthylazine-desethyl | 2.30 | 327 | 54 | 28.6 |
| Terbutryn | 3.66 | 25 | 74 | 52 |
| Thiodicarb | 1.62 | 22 | 0.39 | 18 |

Table S10: Physico-chemical properties of ionic compounds

| **Ionic pesticides** | **Log Kow** | **Solubility in water (in mg L^-1^ at 20°C)** | **DT50 (lab in days)** | **DT50 (field in days)** |
| --- | --- | --- | --- | --- |
| 2.4-D | -0.82 | 24300 | 4.4 | 28.8 |
| Acetochlor-ESA | *n.d.* | *n.d.* | 75.8 | *n.d.* |
| Acetochlor-OA | *n.d.* | *n.d.* | 38.9 | 12 |
| Bentazone | -0.46 | 7112 | 20 | 7.5 |
| Chlorsulfuron | -0.99 | 12500 | 51.4 | 36.2 |
| Dichlorprop | 0.67 | 672 | 12 | 12 |
| Diclofop | 1.61 | 122700 | 23.9 | 35.2 |
| Fenoprop | 3.8 | 140 | *n.d.* | *n.d.* |
| Iodosulfuron | 1.6 | 25000 | *n.d.* | *n.d.* |
| Ioxynil | 2.2 | 3034 | 2.3 | 5 |
| MCPA | -0.81 | 29390 | 24 | 25 |
| Mecoprop | -0.19 | 250000 | 5.24 | 21 |
| Mesotrione | 0.11 | 1500 | 19.6 | 5 |
| Metolachlor-ESA | -1.89 | 212461 | 235 | *n.d.* |
| Metolachlor-OA | *n.d.* | 360000 | 325 | *n.d.* |
| Metsulfuron-methyl | -1.87 | 2790 | 23.2 | 13.3 |
| Nicosulfuron | 0.61 | 7500 | 16.4 | 19.3 |
| Sulcotrione | -1.7 | 165 | 25.3 | 3.6 |

**Detection frequency and maximum TWAC of pesticides/metabolites**

For each sampling site, the detection frequencies of the different pesticides (neutral and ionic compounds) and their highest TWAC values are presented in Fig. S4. On the x axis, pesticides are separated according to their chemical class: neutral pesticides are shown on the left and ionic pesticides on the right.

The detection frequencies support the results described in the manuscript. The detection frequencies were higher for neutral than for ionic pesticides. Neutral pesticides were often detected and constitute a background noise, whereas ionic pesticides were less frequently detected.

A wider variety of pesticides was detected/quantified on the Aixette downstream and upstream sampling sites, with 42 and 38 pesticides, respectively. The Aixette source sampling site was less contaminated, with 19 detected/quantified pesticides.

Overall, 38 out of 64 neutral pesticides and 9 out of 18 ionic pesticides were detected/quantified on at least one deployed POCIS throughout the 3 years.

Regarding the frequency, only neutral pesticides were detected/quantified with frequencies higher than 55% (3, 9, 11 and 6 neutral pesticides were detected/quantified with frequencies higher than 55% for the Aixette source, upstream, downstream and Arthonnet tributary, respectively). Regarding the ionic compounds, no compound was detected with a frequency higher than 50% (except for bentazone in the tributary Arthonnet, with a detection frequency of 55%).

Irrespective of the sampling site, only one compound (DEA – metabolite of atrazine) was detected with a frequency of 100%. Atrazine and diuron were detected, at all sampling sites, at frequencies above 90% (atrazine and diuron).

Regarding the neutral pesticides, 8 pesticides (atrazine, carbofuran, chlortoluron, DEA, imidacloprid, metazachlor, metolachlor and tebuconazole) were detected/quantified at all four sampling sites, whereas 17 pesticides were detected/quantified in at least three sampling sites.

The ionic pesticides-metabolites 2,4-D, dichlorprop, diclofop, MCPA, metolachlor-ESA and metolachlor-OA were detected/quantified at all four sampling sites, whereas six ionic pesticides out of the nine ones detected were commonly found at the four sampling sites. The three other ionic pesticides (bentazone, chlorsulfuron and mecoprop) were detected/quantified at three sites.

Regarding the maximum TWAC, maximum average concentrations were obtained for ionic pesticides. Ionic pesticides have a higher affinity to water than neutral compounds, which explains these results. Metolachlor-OA (ionic metabolite of metolachlor) was quantified at 393 ng L^-1^; it showed the maximum TWAC and was detected in Aixette upstream. Irrespective of the sampling site, these two compounds were quantified at high TWAC levels. In a previous study, S-metolachlor and its metabolite (metolachlor-OA) were detected/quantified in freshwater at high concentrations (De Oliveira Arias et al. 2017).

For neutral pesticides, the maximum TWAC was obtained for the Arthonnet tributary sampling site for dimethenamid, with a TWAC of 191 ng L^-1^. The average maximum TWAC for neutral pesticides was approximately 15 ng L^-1^, whereas the average maximum TWAC for ionic pesticides was approximately 100 ng L^-1^.

The concentrations obtained in this study were average concentrations (TWAC) obtained with POCIS devices. In small streams, studies have shown peaks of pesticides lasting for 1 to 4 days, likely due to the periodic use of pesticides (Kreuger and Törnqvist 1998; Liess et al. 1999). This raises the question of the concentrations that could have been obtained via grab sampling during contamination peaks, especially for ionic pesticides. The monitoring of neutral pesticides by POCIS is adapted and allows pre-concentration *in situ* and the determination of the background noise. The concentration obtained *via* grab sampling might have been higher (Schäfer et al. 2008; Guibal et al. 2018). In future studies, the effects on aquatic organisms, particularly important in headwater streams such as the Aixette Watershed, should be investigated.

Analysis of the contaminants detected/quantified in the water have revealed widespread contamination by various pesticides and their degradation products. The most frequently detected compounds were mainly to the herbicide classes (62%), with a marked presence of certain persistent metabolites (16%).

Atrazine, although banned in several countries including the European Union, was detected and quantified and their persistent metabolites (DIA and DEA). These compounds were result from historical pollution and release from soils, where they remain adsorbed for long periods. Other compounds recognized as persistent like herbicides (*i.e.* diuron, ethidimuron, simazine ...) insecticides (imidacloprid) or fungicides (*i.e.* carbendazim, cyproconazole ...), banned several years ago, was also detected or quantified.

S-metolachlor, an herbicide widely used in maize cultivation, is highly persistent in the environment in the form of its ESA and OXA derivatives, which was detected at high concentrations.

The contamination observed in the analyses was mainly the result of agricultural practices, with a contribution from historical pollution and non-agricultural uses such as rail track weed control, with the presence of ethidimuron. The persistence of certain metabolites showed that even substances banned several years ago can continue to impact water resources.

Analysis of temporal trends in neutral compounds (pesticides and their neutral metabolites) has revealed a seasonal pattern in water contamination (Fig. 2). A slight variation in concentrations is observed over all the years studied, with marked peaks at the end of spring. Periods of pic contamination was coincided with the seasons of pesticide application in agricultural settings, particularly spring (herbicide and fungicide treatments) and summer (insecticide and fungicide treatments). These observations are in line with previous studies (Guibal et al. 2018) which have shown a correlation between phytosanitary practices and residue detected in surface. Conversely, the winter period was marked by a general decrease in concentrations, which could be attributed to a combination of reduced pesticide use, adsorption in the soil and increased dilution by precipitation. As mentioned in the manuscript, ionic compounds showed more pronounced fluctuations than neutral compounds, suggesting greater mobility and sensitivity to hydrological and climatic conditions.

Finally, inter-annual analysis of pesticides contamination (neutral and ionic) has revealed a recurrence of seasonal trends, although variations in peak intensity can be observed from year to year. These differences could be linked to climatic factors (rainfall, temperature) or changes in farming practices. These temporal dynamics underline the need for continuous monitoring and adaptive management of pesticides, integrating the effects of seasons and climatic events.

*Figure S5: Pesticide detection frequencies (grey) and highest TWAC (ng L^-1^) (in orange for highest TWAC and green for detection below the limit of quantification) determined by POCIS-HLB and POCIS-MAX during 2017, 2018 and 2019 and for A: Aixette source (nPOCIS-HLB = 61 and nPOCIS-MAX = 58), B: Aixette upstream (nPOCIS-HLB = 77 and nPOCIS-MAX = 76), C: Aixette downstream (nPOCIS-HLB = 72 and nPOCIS-MAX = 72) and D: Arthonnet tributary (nPOCIS-HLB = 73 and nPOCIS-MAX = 75). Metabolites are identified with an asterisk.*

**References**

De Oliveira Arias JL, Schneider A, Batista-Andrade JA, et al (2017) Evaluation of dilute-and-shoot and solid-phase extraction methods for the determination of: S -metolachlor and metolachlor-OA in runoff water samples by liquid chromatography tandem mass spectrometry. Analytical Methods 9:5777–5783. https://doi.org/10.1039/c7ay01698k

Guibal R, Lissalde S, Leblanc J, et al (2018) Two sampling strategies for an overview of pesticide contamination in an agriculture-extensive headwater stream. Environmental Science and Pollution Research 25:14280–14293. https://doi.org/10.1007/s11356-017-9883-7

Kreuger J, Törnqvist L (1998) Multiple regression analysis of pesticide occurrence in streamflow related to pesticide properties and quantities applied. Chemosphere 37:189–207. https://doi.org/10.1016/S0045-6535(98)00037-X

Liess M, Schulz R, Liess MH-D, et al (1999) Determination of insecticide contamination in agricultural headwater streams. Water Research 33:239–247. https://doi.org/10.1016/S0043-1354(98)00174-2

Schäfer RB, Paschke A, Vrana B, et al (2008) Performance of the Chemcatcher® passive sampler when used to monitor 10 polar and semi-polar pesticides in 16 Central European streams, and comparison with two other sampling methods. Water Research 42:2707–2717. https://doi.org/10.1016/j.watres.2008.01.023
